# Reception of Pathological Specimens

**Published:** 1866

**Authors:** 


Reception of Pathological Specimens.—We have received some valuable additions to our "pathological collection" within the last few weeks, and desire to express our hearthy thanks for these favors. Dr. Charles L. Dayton, North Buffalo, has sent us a specimen of the "Taenia," the patient from whom it was obtained, coming up from New York city to receive his treatment. Dr. Merrill H. Shaw has also presented us with a fœtus, born at full time, with *extroverted brain*, the cerebral mass laying over the dorsal region below the neck, or below where the neck should be. It is not covered by skull or integuments, but contained in the membranes proper to the brain. Dr. W. McCarjow, of Caledonia, C.W., has sent a very interesting and instructive specimen of disease of the joint, and also a photograph taken before amputation. We cannot sufficiently express our indebtedness to our professional friends who thus favor us. Our private pathological collection, has grown recently with encouraging and gratifying rapidity, under the generous patronage of friends.

